# Validation of forearm fracture diagnoses in administrative patient registers

**DOI:** 10.1007/s11657-023-01322-x

**Published:** 2023-08-24

**Authors:** Tone Kristin Omsland, Lene B. Solberg, Åshild Bjørnerem, Tove T. Borgen, Camilla Andreasen, Torbjørn Wisløff, Gunhild Hagen, Trude Basso, Jan-Erik Gjertsen, Ellen M. Apalset, Wender Figved, Jens M. Stutzer, Frida I. Nissen, Ann K. Hansen, Ragnar M. Joakimsen, Elisa Figari, Geoffrey Peel, Ali A. Rashid, Jashar Khoshkhabari, Erik F. Eriksen, Lars Nordsletten, Frede Frihagen, Cecilie Dahl

**Affiliations:** 1 Department of Community Medicine and Global Health, Institute of Health and Society, University of Oslo, Blindern, Po box 1130, 0318 Oslo, Norway; 2 Division of Orthopaedic Surgery, Oslo University Hospital, Oslo, Norway; 3 Department of Obstetrics and Gynaecology, University Hospital of North Norway, Tromsø, Norway; 4 Department of Clinical Medicine, The Arctic University of Norway, Tromsø, Norway; 5 Norwegian Research Centre for Women’s Health, Oslo University Hospital, Oslo, Norway; 6 Department of Rheumatology, Vestre Viken Hospital Trust, Drammen Hospital, Drammen, Norway; 7 Department of Orthopaedic Surgery, University Hospital of North Norway, Tromsø, Norway; 8 Health Services Research Unit, Akershus University Hospital, Lørenskog, Norway; 9 Department of Health Services, Norwegian Institute of Public Health, Oslo, Norway; 10 Department of Orthopaedic Surgery, St. Olavs University Hospital, Trondheim, Norway; 11 Department of Orthopaedic Surgery, Haukeland University Hospital, Bergen, Norway; 12 Department of Clinical Medicine, University of Bergen, Bergen, Norway; 13 Bergen Group of Epidemiology and Biomarkers in Rheumatic Disease, Department of Rheumatology, Haukeland University Hospital, Bergen, Norway; 14 Department of Global Public Health and Primary Care, University of Bergen, Bergen, Norway; 15 Department of Orthopaedic Surgery, Vestre Viken Hospital Trust, Bærum Hospital, Gjettum, Norway; 16 Institute of Clinical Medicine, University of Oslo, Oslo, Norway; 17 Department of Orthopaedic Surgery, Møre and Romsdal Hospital Trust, Hospital of Molde, Molde, Norway; 18 Department of Medicine, University Hospital of North Norway, Tromsø, Norway; 19 Pilestredet Park Specialist Centre, Oslo, Norway; 20 Faculty of Dentistry, University of Oslo, Oslo, Norway; 21 Department of Orthopaedic Surgery, Østfold Hospital Trust, Grålum, Norway

**Keywords:** Validation, Forearm fracture, Sensitivity, Positive predictive value

## Abstract

**Summary:**

The validity of forearm fracture diagnoses recorded in five Norwegian hospitals was investigated using image reports and medical records as gold standard. A relatively high completeness and correctness of the diagnoses was found. Algorithms used to define forearm fractures in administrative data should depend on study purpose.

**Purpose:**

In Norway, forearm fractures are routinely recorded in the Norwegian Patient Registry (NPR). However, these data have not been validated. Data from patient administrative systems (PAS) at hospitals are sent unabridged to NPR. By using data from PAS, we aimed to examine (1) the validity of the forearm fracture diagnoses and (2) the usefulness of washout periods, follow-up codes, and procedure codes to define incident forearm fracture cases.

**Methods:**

This hospital-based validation study included women and men aged ≥ 19 years referred to five hospitals for treatment of a forearm fracture during selected periods in 2015. Administrative data for the ICD-10 forearm fracture code S52 (with all subgroups) in PAS and the medical records were reviewed. X-ray and computed tomography (CT) reports from examinations of forearms were reviewed independently and linked to the data from PAS. Sensitivity and positive predictive values (PPVs) were calculated using image reports and/or review of medical records as gold standard.

**Results:**

Among the 8482 reviewed image reports and medical records, 624 patients were identified with an incident forearm fracture during the study period. The sensitivity of PAS registrations was 90.4% (95% CI: 87.8–92.6). The PPV increased from 73.9% (95% CI: 70.6–77.0) in crude data to 90.5% (95% CI: 88.0–92.7) when using a washout period of 6 months. Using procedure codes and follow-up codes in addition to 6-months washout increased the PPV to 94.0%, but the sensitivity fell to 69.0%.

**Conclusion:**

A relatively high sensitivity of forearm fracture diagnoses was found in PAS. PPV varied depending on the algorithms used to define cases. Choice of algorithm should therefore depend on study purposes. The results give useful measures of forearm fracture diagnoses from administrative patient registers. Depending on local coding practices and treatment pathways, we infer that the findings are relevant to other fracture diagnoses and registers.

## Introduction

Register data are frequently used for research, surveillance of disease, and planning of healthcare services. However, hospital-based register data are collected for administrative purposes, which affects the type of data available and data quality [1]. The quality of register data varies depending on the data source, diagnosis, and year [2, 3]. It is recommended that validation studies are performed before using register data for research [4].

Compared to the UK, Sweden, and Australia, a high incidence rate of forearm fractures has been reported in Oslo, Norway, where the diagnoses were verified by manually reviewing medical records and X-ray reports [5]. Only one study has been published on forearm fractures in the Norwegian Patient Registry (NPR), but no validation of the diagnoses was performed [6]. On the other hand, hip fracture diagnoses in the NPR have been extensively studied [7, 8] and validated [9]. Hip fracture patients are routinely treated surgically and thereby better captured in the hospital-based registries. In contrast, only 30% of forearm fractures are surgically treated in Norway, with a variation between hospitals of 16–40% [6]. Hence, forearm fractures are more challenging to capture in register data than hip fractures.

Most forearm fractures in Norway are treated at hospitals, but some fractures may be treated in primary care only (e.g., in rural areas with a decentralized X-ray service). In a study comparing data from NPR and the primary health care register, 93% of forearm fractures were captured by NPR whereas 7% of all forearm fracture registrations were identified only in primary care (after wash-out and exclusion of records coded as follow-up visits) [10]. This is probably an overestimate, because some diagnoses in primary care represents suspected fractures that could not be separated from true fracture events. Medical record review at five primary care facilities located far from hospitals found that 60% of the forearm fracture registrations were incident [10], but it is not known whether this figure is representative for all primary care facilities treating forearm fractures in Norway. The background for this validation study is the Norwegian Capture the Fracture Initiative (NoFRACT), a multi-center study at seven hospitals in four health regions of Norway, that investigated the effect of a fracture liaison service (FLS) on subsequent fragility fracture rates [11]. The outcome data in NoFRACT will be obtained from NPR and consequently there is a need for validation of hospital data.

Incorrect medical coding can lead to both over- and under-reporting of specific diagnoses in administrative data. Another challenge in register-based research of fractures is to correctly define incident versus prevalent cases when the same patient has multiple registrations of the same diagnosis code [12–14]. To handle this, it is common to introduce a washout period, which means that a patient can only count once within a specific time window. However, other logged information can also be used to improve the correctness of the administrative data, such as procedure codes, whether the S52 code is the main or additional diagnosis, and follow-up codes [12, 13].

The aim of this study was to examine the sensitivity and positive predictive value (PPV) of the forearm fracture diagnosis (S52) in Patient Administrative Systems (PAS) at five hospitals, using X-ray reports and/or medical record reviews as the gold standard. In addition, we wanted to examine the optimal washout period for identification of incident forearm fractures, and the usefulness of procedure codes and follow-up codes to improve the data quality.

## Material and methods

### Treatment of forearm fractures in Norway

In Norway, most patients with forearm fracture are treated at hospitals which report data to the NPR. These data are widely used for research [15]. In the larger cities, there are hospital-affiliated outpatient units that also report data to the NPR. However, approximately 7% of forearm fractures are treated in primary care only and therefore not reported to the NPR [10]. It is mandatory for the hospitals to report monthly about patients treated at hospitals and emergency units to NPR. Data are sent unabridged from PAS to NPR [16], and hospital discharge data can therefore be used as a replacement for NPR data, minimizing the transfer of data between institutions.

### Data from patient administrative systems at the hospitals

The Norwegian Capture the Fracture Initiative (NoFRACT) is a multi-center study including patients treated at seven hospitals in four health regions of Norway [11]. Patients aged ≥ 19 years treated for a forearm fracture were manually retrieved from PAS at five of the seven NoFRACT hospitals in the following assigned periods: Oslo University Hospital, Ullevål 2 Feb–1 Mar 2015, Drammen Hospital 2 Feb–22 Mar 2015, Bærum Hospital 2 Feb–26 Apr 2015, University Hospital of North Norway, Tromsø 2 Feb–26 Apr 2015, and Molde Hospital 2 Feb–20 Sept 2015. The periods were tailored to obtain approximately 200 forearm fracture registrations from each hospital. The periods will hereafter be referred to as “the study period.”

**Fig. 1:**
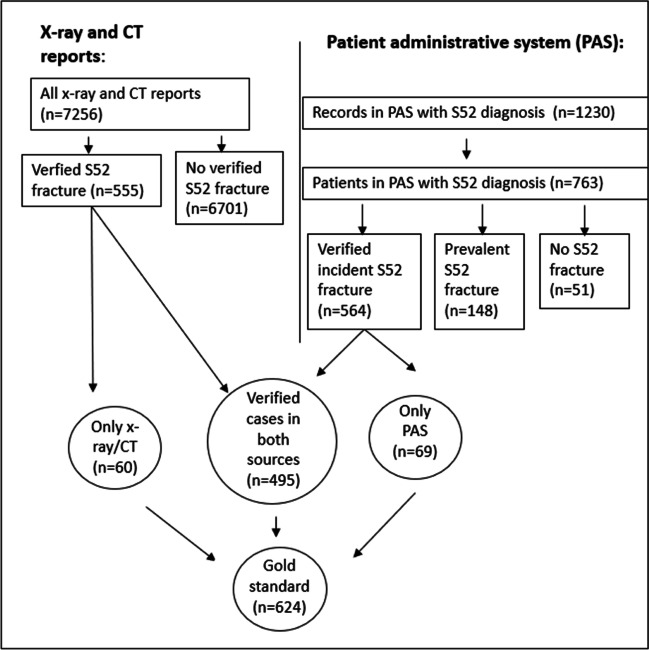
Flow diagram of data in the study of forearm fracture diagnoses (S52) from image reports and the patient administrative system (PAS) in five hospitals

Patients were identified in PAS by a search for ICD-10 codes S52 with all sub-groups (forearm fractures) and S62.8 (fracture of other and unspecified parts of wrist and hand) (Fig. 1). None of the patients with an S62.8 registration (*n* = 4) had an incident forearm fracture, and they were excluded from further analyses. We collected patient-level information using the national identity number; age, sex, whether the fracture was coded as a main or additional diagnosis, date of examination, date of discharge, record date, name of the first treatment unit (if sustained in Norway), date of surgery (if applicable), procedure-codes, and ICD-10 diagnosis codes indicating follow-up visits.

### Data from conventional X-ray and CT reports

Radiological data was obtained independently of the PAS data. This data source was extracted from data systems at the departments of radiology at each hospital, using radiologic codes and keywords from both CT and conventional X-ray. All CT and conventional X-ray examinations of the upper extremities were included in our search (except fingers). To capture delayed registration and identify fractures sustained before the study period, conventional X-ray and CT reports were retrieved within the assigned study periods ± 1 week. The Norwegian Classification of Radiological Procedures (NCRP) has been used since 2012, and we planned to search for the NCRP codes for examinations of the hand, wrist, forearm, elbow, and upper arm. However, the coding of these NCRPs were incomplete and additional searches therefore included the keywords “forearm,” “wrist,” “hand,” “scaphoid,” and “upper extremity.”

All reports were manually checked to verify whether the patient sustained (1) an incident forearm fracture within the study period, (2) a prevalent forearm fracture before the study period, (3) no fracture, or (4) other type of fracture. Information on the national identity number, age, sex, date of the X-ray/CT, and reports were obtained.

### Combining data from X-ray and medical records—defining the “gold standard”

The data from PAS and from the image reports were merged by the national identity number. The gold standard in this study was defined as all incident forearm fractures verified from X-ray/CT reports or review of medical records. In cases where X-ray reports were missing or the information was inconclusive, fractures were verified by an additional review of the medical records. In cases where the image reports were unclear, the results were compared to medical records, where a fracture could be clinically verified or excluded. In cases where no medical records were available and the outcome was inconclusive (*n* = 13), orthopedic surgeons and clinicians were consulted.

### ICD-10 diagnosis codes and surgical procedure codes

Registrations in PAS were categorized as either main or additional diagnosis. The ICD-10 codes Z09.4 (follow-up of fracture), Z09.0 (follow-up non-cancer), Z09.8 (follow-up for specified diagnosis), Z46.7 (follow-up orthopedic equipment), Z47.0 (follow-up, removal of osteosynthesis material), Z47.8 (follow-up, removal or revision of casting or fixation), Z88.8 (follow-up, complications after surgery), T92.1 (follow-up, sequela of fracture of arm), and T92.2 (follow-up, sequela of wrist and hand) were considered “follow-up code” (Table 1).

**Table 1:** Codes and criteria for diagnosis, washouts, and procedures used to create different algorithms to define forearm fracture cases in patient administrative system

Term or code	Explanation
Crude cases	All patients with a S52 code within the study period were included
Washout (3, 6, or 12 months)	Prevalent S52 cases 3, 6, or 12 months prior to the registration were omitted
Main diagnosis	Only records with S52 coded as main diagnosis were included
Procedure codes^a^	Only patients with codes indicating incident fracture were included
Follow-up codes	Records with follow-up codes were excluded

^a^Procedure codes with all subgroups were included. The surgical procedure codes NCJ, NDJ, TND, and TNC were used

We used the NOMESCO (Nordic Medico-Statistical Committee) Classification of Surgical Procedures (NCSP) groups of codes, NCJ (Fracture surgery of elbow and forearm), NDJ (Fracture surgery of wrist and hand), TND (Minor procedures in wrist and hand), and TNC (Minor procedures in elbow and forearm) with all subgroups.

### Statistical analyses

Sensitivity was calculated by dividing the number of verified incident forearm fractures identified in PAS (“true positives”) by the gold standard. Positive predictive value (PPV) was calculated by dividing the number of “true positives” by the total number of patients identified in PAS with S52 codes. Sensitivity and PPV with 95% confidence intervals were calculated by using the STATA module “diagti.”

The sensitivity and PPV by different aspects of coding practice were investigated including timing of registrations, and combinations of codes (Table 1). For the crude comparison of fracture registrations in PAS versus the gold standard, all forearm fracture registrations in PAS which were found to be prevalent fractures according to the gold standard, were coded as “no fracture.” We performed separate calculations where prevalent registrations within a period of 3, 6, and 12 months prior to the PAS registration were omitted from the numerator and the denominator, whereas the PAS registrations prior to the washout were coded as “no fracture.” The rationale for doing this is that washout in register data means that patients are only allowed to count once within a specified period.

Microsoft Excel 2016 (Microsoft, Redmond, WA, USA) was used for data collection and Stata 16 (StataCorp, College Station, TX, USA) for data cleaning, merging, and analysis.

## Results

### Verified incident forearm fractures—gold standard

A total of 7256 image reports from 5440 patients were retrieved from the five hospitals, and 555 of these patients had a verified forearm fracture (Fig. 1). A total of 624 patients with incident forearm fractures were verified either by image reports or medical records, the gold standard. There were 6 patients with simultaneous bilateral forearm fractures registered on the same day. No other patients had more than one forearm fracture event during the study period. Women constituted 73% of the patients, and 77% of the fractures were located at the distal forearm (Table 2). The median age at fracture was 62 years [interquartile range (IQR): 52–73] in women, and 47 years [IQR: 33–64] years in men.

**Table 2:** Distribution of the verified incident forearm fracture subtypes in 624 patients at five Norwegian hospitals

Fracture subtype^a^	*n*	%
S52.0 Fracture of upper end of ulna	25	4
S52.1 Fracture of upper end of radius	49	8
S52.2 Fracture of shaft of ulna	6	1
S52.3 Fracture of shaft of radius	14	2
S52.4 Fracture of shafts of both ulna and radius	0	0
S52.5 Fracture of lower end of radius	481	77
S52.6 Fracture of lower end of both ulna and radius	29	5
S52.7 Multiple fractures of forearm	1	0
S52.8 Fracture of other parts of forearm	18	3
S52.9 Fracture of forearm, part unspecified	1	0

^a^Verified by the gold standard (x-ray and CT report and/or medical records)

### Incident forearm fractures in PAS

A total of 1230 forearm fracture diagnose in 763 patients were registered in PAS (Fig. 1). Of these 763 patients, 564 had a verified incident forearm fracture, 148 had a prevalent forearm fracture, and 51 had no forearm fracture (Fig. 1). A total of 499 patients had one registration, while the remaining patients had between two and 10 registrations of a forearm fracture in the study period. The length of the study period (range 27–230 days) and number of patients (range 112–196) varied according to size of the hospital (Table 3).

**Table 3:** The study period, total number of registrations of S52 diagnoses and patients in the patient administrative system (PAS) at five hospitals

			Patients		
	Period	Registrations	Main diagnoses		Total
	(days)	(*n*)^a^	(*n*)	(%)	(*n*)
Bærum	83	203	138	98	141
Drammen	48	250	122	72	169
Molde	230	200	104	93	112
Oslo	27	234	192	98	196
Tromsø	83	343	126	87	145
Total	471	1230	682	89	763

^a^Total number of registrations in PAS

Regarding patients with verified fractures in PAS, the days between the PAS registration and the verified date ranged between 0 and 92 days; 80.8% were registered on the same day, 90.4% within 14 days and 95.1% within 30 days.

### Sensitivity and positive predictive values

PAS covered 564 of the 624 verified incident forearm fractures and the sensitivity of the forearm fracture diagnosis was 90.4% (95% CI 87.8–92.6). Among 60 patients with verified fractures not recorded in PAS, 9 had their primary treatment in another hospital. Other reasons for missing in PAS included miscoding (*n* = 1), external referral (*n* = 2), and that the fracture diagnosis was not verified until the second visit (*n* = 3). There were 6 patients with undetermined date of fracture (possibly prevalent fractures). In 39 patients, we were not able to find the reasons why they were missing in PAS. The highest proportion of unexplained missing registrations in PAS was found at the hospital in Tromsø (*n* = 13) where several remote locations have X-ray available in primary care, and digitally transfer images to the hospital for consultation.

The 564 of 763 patients with verified incident forearm fracture in PAS, resulted in a crude PPV of 73.9% (95% CI: 70.6–77.0) (Table 4). The main reason for miscoding (*n* = 199) was that 148 (74%) of the patients had a verified prevalent S52-fracture (Table 4). Most of the prevalent fractures (*n* = 136, 92%) were sustained during the 3 months before, whereas the remaining 12 were sustained more than 3 months before the study period. In 51 miscoded registrations, the patients had other fractures (S42, S62, S72, S82, S92), or other injures but no incident forearm fracture verified by X-ray and CT report review or in medical records.

**Table 4:** Type of miscoding of 199 registrations of S52 diagnosis codes in the patient administrative system at five hospitals, not verified as incident forearm fracture by x-ray or medical records

Miscoded registrations	*n*	(%)
Fracture of shoulder and upper arm (S42)	3	2
Fracture at wrist and hand level (S62)	11	6
Fracture of femur (S72)	2	1
Fracture of lower leg, including ankle (S82)	4	2
Fracture of foot, except ankle (S92)	1	1
Other injuries (suspected fracture, strain, sprain etc.)	30	15
Prevalent fractures (sustained before study period)	148	74
Total	199	100

In a sensitivity analysis with 6 months washout, we excluded 6 patients with uncertain date of fracture and 4 patients with uncertain fracture status and the sensitivity was 91.3% (95% CI: 88.8–93.4%) and PPV = 91.0% (95% CI: 88.4–93.1%).

### Use of washout periods for PPV calculation

As shown in Table 5, washout periods of 3, 6, or 12 months separated cases with prevalent from incident forearm fractures similarly. Eight prevalent fractures were sustained more than 6 months before the PAS entry date, and if including a washout period of 6 months, we would categorize the 140 prevalent fractures correctly, increasing the PPV of the incident S52 fractures from 73.9 to 90.5% (Table 5).

**Table 5:** Positive predictive value (PPV) using different washout periods to define incident forearm fractures in the patient administrative system (PAS)

	Registrations in PAS^a^	Verified fractures^b^	Miscoded^c^	PPV
Crude comparison	763	564	199	73.9
3-month washout	627	564	63	90.0
6-month washout	623	564	59	90.5
12-month washout	622	564	58	90.7

^a^Registrations in PAS (without prevalent fractures during washout)

^b^Incident fractures verified by X-ray/CT or medical journal review

^c^Sum of miscoded registrations and prevalent fractures with different washout criteria

### Additional use of follow-up codes and procedure codes for PPV calculation

**Fig. 2:**
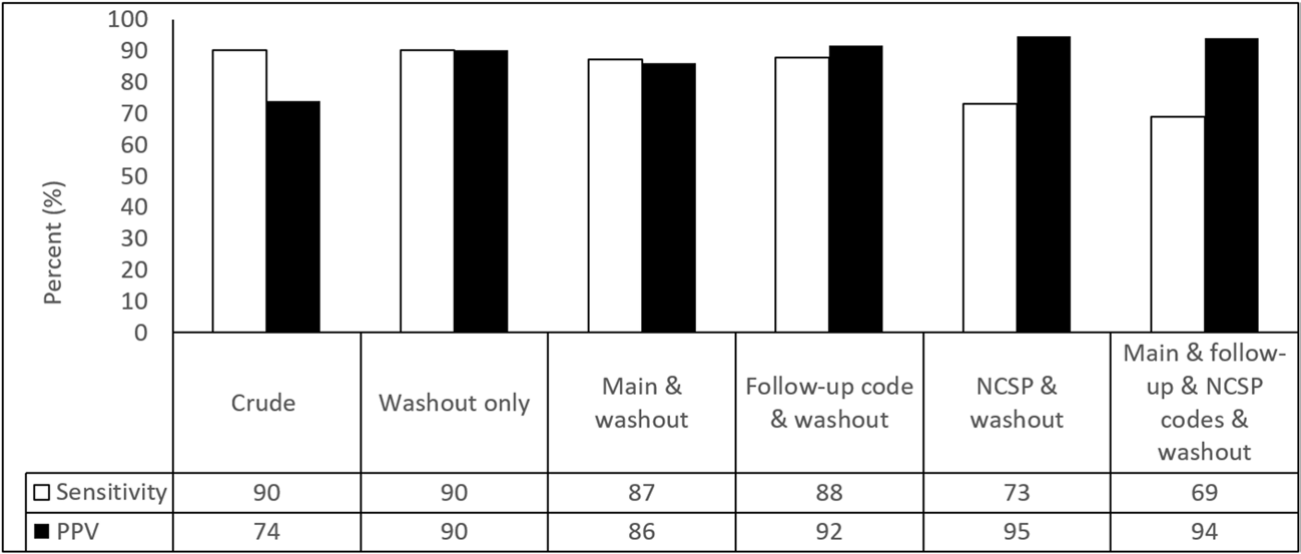
Estimated sensitivity and positive predictive value (PPV) by different algorithms to define forearm fracture cases (algorithm details in Table 1)

Among 564 patients with a verified incident fracture in PAS, 16 patients (2.8%) had registered follow-up codes. In records with S52 diagnoses verified as prevalent fractures, 62.4% had a follow-up code. Using follow-up codes in addition to 6 months washout increased the PPV from 74 to 88% (Fig. 2).

In 682 patients with S52 as the main diagnosis, 6% had no verified incident S52 fracture. Of 763 registrations with S52 codes in PAS, 682 (89%) were registered as the main diagnosis code and 81 (11%) as an additional diagnosis code (Table 3). Of these 81 diagnoses, 25 (31%) had an incident S52 fracture, 47 (58%) had a prevalent S52 fracture, and 9 (11%) had no verified S52 fracture. Among the 25 patients with incident S52 fractures as additional diagnosis, 12 (48%) patients had been to a primary care physician, were treated outside the hospital, or sustained the fracture abroad. Excluding patients with fracture as an additional diagnosis reduced the sensitivity and the PPV slightly (Fig. 2). Additional use of follow-up codes increased the PPV more than washout only but reduced the sensitivity.

There were 297, 69, and 123, patients with a TND, TNC, or NCJ code, whereas no patients had an NDJ procedure code. A total of 453 (80.3%) of the verified fractures in PAS had one or more of the procedure codes during the study period. Additional use of surgical procedure codes increased the PPV more than washout alone, but the sensitivity was lower. Using all available codes gave excellent PPV, but the sensitivity was reduced (Fig. 2).

## Discussion

In this study, forearm fracture registrations at five Norwegian hospitals were validated using reports from either X-ray, CT,or medical records as the gold standard. The sensitivity was 90% and the crude PPV of 74% increased to 90% by using a washout period of 6 months. Registration of follow-up codes and procedure codes was incomplete, and coding practices varied between the hospitals. The PPV increased when using a combination of washout period of 6 months, follow-up codes, and procedure codes but at the expense of the sensitivity.

To the best of our knowledge, a validation of forearm fracture diagnosis on a national level has not been performed in Norway or any other Scandinavian country. However, a subset of 471 incident distal radius fractures in the Skåne Health Care register in Sweden, was validated by reviewing medical records as the gold standard [17]. The sensitivity of the register data was 90% and PPV 94%. Our results of 90% sensitivity and PPV are in line with the findings, although our PPV was somewhat lower. In a population-based validation study of humeral fractures in the Danish National Patient Registry (DNPR) in 2017–2020 [18], the PPV was 89.3%, whereas another study of all orthopedic diagnoses in the DNPR in a 2-week period in 2006 reported a PPV of 86% [19]. In a validation study of 1000 hip fracture patients in Norway, the PPV was 98.2% [9]. Likewise, a study from the Finish patient register found that 98.1% of hip fractures had a hip fracture diagnosis [20]. The lower validity of non-hip fractures probably reflects that other fractures are more challenging to capture due to their diversity in treatment, place of treatment, and that hip fractures are routinely treated surgically.

In order to obtain estimates near the true value when calculating incidence rates, it is advantageous if the false negative and the false positive cases balance out [1]. For studies of incidence rates of forearm fracture, the best combination of sensitivity and PPV was found with 6-month washout only. On the other hand, if the data are to be used for other purposes, for example when studying an association in a cohort study, it might be more appropriate to use the algorithm that produced the highest PPV. Although the results for 3- and 6-month washout were similar, it is reasonable to use a 6-month washout as both treatment and fracture healing usually are terminated by then.

The PPV is a valid measure of validity in the current study, but it only covers one dimension of registration practice. Estimating sensitivity is challenging in this type of study as it ideally would need to cover all patients seeking healthcare treatment during the study period [21]. This was not feasible, and the sensitivity might be overestimated. The proportion of patients with miscoded S52 registrations (verified non-arm fractures) was 0.9%, and the proportion of true S52 fractures miscoded as non-arm fractures is likely to be low. Another weakness regarding the sensitivity calculations is that some patients with subsequent forearm fractures in register data will only be counted once when introducing a washout period and that was not accounted for in our calculations. However, a study from Iceland of 2364 medical records verified incident forearm fractures reported that 11% of patients with a first forearm fracture sustained a second forearm fracture within 10 years [22]. Given a similar risk of subsequent forearm fractures in Norway, we would exclude approximately 1.5% of incident forearm fractures when using a 6-month washout period. Hence, the sensitivity after 6-month washout is likely to be 1.5% lower than the estimated 90.4% that we reported in this study. On the other hand, in the current study, nine of the patients not found in PAS (1.4%) were treated in other hospitals and would probably be captured if using data from the NPR. Although data from PAS is said to be transferred unabridged to the NPR, some minor data processing seems to take place when combining data from all Norwegian hospitals. Future studies should compare hospital data with data from NPR (collected from hospitals) on an individual level to obtain direct information about the data from NPR.

A strength of this study is that we reviewed more than 8000 medical records and X-ray and CT reports from five hospitals in Norway in 2015 and estimated both sensitivity and PPVs. We included data from only five of 44 hospitals treating forearm fractures in Norway. Still, the data are likely to be representative for the country as the included hospitals vary in size and are located in all four health regions of Norway. Changes in classification systems and the use of more sensitive diagnostic methods over time (diagnostic drift) may hamper the interpretation of secular trends in incidence rates. A limitation is that we only validated data from the year 2015 and were unable to investigate any changes in medical coding practice over time. It would have been useful to have data from three or more years, but due to methodologic challenges and limited resources this was not possible.

## Conclusion

The data from the patient administrative system from five hospitals in Norway showed a sensitivity of approximately 90% and the PPV varied between 74 and 95% depending on the algorithm used to define incident forearm fractures. The results are valuable measures of the validity of forearm fracture diagnoses obtained from administrative patient registers. Depending on local coding practices and treatment pathways, our estimates are relevant for other fracture diagnoses and research based on register data in other countries.

## Data Availability

Availability of data depend on permissions from the Norwegian Directorate of Health, evaluation by the Norwegian Centre for Research Data, and approval from the data protection officer at the University of Oslo and at the hospitals. The data collected in this study were not available to other investigators or data managers than those included in project description (listed in the data protection impact assessment). The investigators at the hospitals who collected data were only able to upload data and not access any other data in the safe research platform at the University of Oslo. Access to data for other investigators can be obtained after approval from Norwegian Directorate of Health, evaluation by the Norwegian Centre for Research Data, and approvals from the data protection officers at the hospitals and the University of Oslo.
